# Fertility specialists’ views, behavior, and attitudes towards the use of endometrial scratching in Italy

**DOI:** 10.1186/s12905-023-02564-0

**Published:** 2023-07-29

**Authors:** Stefano Palomba, Domenico Carone, Amerigo Vitagliano, Flavia Costanzi, Alice Fracassi, Tiziana Russo, Serena Del Negro, Altiero Biello, Aldo Di Filippo, Antonio Mangiacasale, Antonio Monaco, Antonio Ranieri, Beatrice Ermini, Bruno Francesco Barba, Claudio Castello, Federica Di Guardo, Francesco Pastorella, Elena Bernasconi, Ezio Michele Tricarico, Francesca Filippi, Francesco Polsinelli, Giuseppe Lo Monte, Loredana M. Sosa Fernandez, Marco Galletta, Paolo Giardina, Pasquale Totaro, Roberto Laganara, Roberto Liguori, Matteo Buccheri, Mario Montanino Oliva, Rosita Piscopo, Assunta Iuliano, Nicola Innantuoni, Irene Romanello, Francesco Sinatra, Annalisa Liprino, Roberto Thiella, Alessandra Tiezzi, Tiziana Bartolotti, Alessandra Tomasi, Valeria Finocchiaro, Mario Thiella, Giuseppa Fuggetta, Sebastiano Messineo, Francesco Isabella, Marcello Tripodi, Stefania Iaccarino, Giovanni Battista La Sala, Enrico Papaleo, Donatella Caserta, Roberto Marci, Edgardo Somigliana, Antonino Guglielmino, the Italian Society of Human Reproduction (SIRU)

**Affiliations:** 1 Sant’Andrea Hospital, University “Sapienza” of Rome, Rome, Italy; 2 Eugin, Taranto, Italy; 3 Centro Di PMA, Ospedale Di San Donà Di Piave, Venice, Italy; 4 Nike Medical Center, Rome, Italy; 5 Grande Ospedale Metropolitano, Reggio Calabria, Italy; 6 Gatjc Fertility Center, Gioia Tauro, Reggio Calabria, Italy; 7 Presidio Ospedaliero Di Soverato “Basso Ionio”, Soverato, Catanzaro Italy; 8 ASL Na2 Nord, Naples, Italy; 9 Centro Di Riproduzione Assistita - CRA, Catania, Italy; 10 Promea, Turin, Italy; 11 Clinica HERA, Giugliano in Campania, Naples, Italy; 12 Centro Italiano Di Procreazione Assistita - CIPA, Rome, Italy; 13 Casa Di Cura “Salus”, Brindisi, Italy; 14 Centro FIVET Città Di Torino, Casa Della Salute Valdese, Turin, Italy; 15 Azienda Ospedaliero Universitaria Policlinico “G. Rodolico – San Marco”, Catania, Italy; 16 01 Procreazione: Centro Di Fecondazione Assistita, Mestre, Venice, Italy; 17 Clinica San Carlo, Paderno Dugnano, Milan, Italy; 18 Centro Di PMA, Ospedale Di Nardò, Lecce, Italy; 19 Fondazione IRCCS Ca’ Grande – Ospedale Maggiore – University of Milan, Milan, Italy; 20 Centro STS, Sora, Frosinone, Italy; 21 Centro Di Medicina Della Riproduzione E Crioconservazione Dei Gameti, Ospedale Di Brunico, Bolzano, Italy; 22 Centro Embryos, Battipaglia, Naples, Italy; 23 Centro Di Procreazione Medicalmente Assistita, Azienda Ospedaliera “Papardo”, Messina, Italy; 24 IRCCS San Raffaele Hospital, Milan, Italy; 25 Centro Di Procreazione Medicalmente Assistita, Ospedale Santa Maria, Bari, Italy; 26 Biotech PMA, Padua, Italy; 27 Poliambulatorio Vismed, Bari, Italy; 28 Genersi Srl - Istituto Bernabeu Venecia, Venice, Italy; 29 Altamedica, Rome, Italy; 30 ASL 5 Spezzino, La Spezia, Italy; 31 UOC Di Ostetricia E Ginecologia, Azienda Ospedaliera “San Carlo”, Potenza, Italy; 32 Centro Chemis, Naples, Italy; 33 SSD Di Procreazione Medicalmente Assistita, Azienza Sanitaria Friuli Occidentale, Sacile, Pordenone Italy; 34 Azienda ULSS 7 Pedemontana, Bassano del Grappa, Vicenza, Italy; 35 Unità Di Medicina Della Riproduzione - UMR, Catania, Italy; 36 Poliambulatorio Teatro Nuovo, Vicenza, Italy; 37 Clinica Nuova Ricerca, Rimini, Italy; 38 Centro Demetra-Artebios, Lugo, Ravenna, Italy; 39 Ospedale San Luca, Trecenta, Rovigo, Italy; 40 Centro Fertilia, Casa Di Cura “Falcidia”, Catania, Italy; 41 Clinical Institute of Sterility, Lamezia Terme, Catanzaro, Italy; 42 Network 9.Baby, Bologna, Italy; 43 University of Ferrara, Ferrara, Italy

**Keywords:** Endometrial injury, Endometrial scratching, Infertility, IVF, Sterility

## Abstract

**Background:**

Endometrial scratching (ES) or injury is intentional damage to the endometrium performed to improve reproductive outcomes for infertile women desiring pregnancy. Moreover, recent systematic reviews with meta-analyses and randomized controlled trials demonstrated that ES is not effective, data on the safety are limited, and it should not be recommended in clinical practice. The aim of the current study was to assess the view and behavior towards ES among fertility specialists throughout infertility centers in Italy, and the relationship between these views and the attitudes towards the use of ES as an add-on in their commercial setting.

**Methods:**

Online survey among infertility centers, affiliated to Italian Society of Human Reproduction (SIRU), was performed using a detailed questionnaire including 45 questions with the possibility to give “closed” multi-choice answers for 41 items and “open” answers for 4 items. Online data from the websites of the infertility centers resulting in affiliation with the specialists were also recorded and analyzed. The quality of information about ES given on infertility centers websites was assessed using a scoring matrix including 10 specific questions (scored from 0 to 2 points), and the possible scores ranged from 0 to 13 points (‘excellent’ if the score was 9 points or more, ‘moderate’ if the score was between 5 and 8, and ‘poor’ if it was 4 points or less).

**Results:**

The response rate was of 60.6% (43 questionnaires / 71 infertility SIRU-affiliated centers). All included questionnaires were completed in their entirety. Most physicians (~ 70%) reported to offer ES to less than 10% of their patients. The procedure is mainly performed in the secretory phase (69.2%) using pipelle (61.5%), and usually in medical ambulatory (56.4%) before IVF cycles to improve implantation (71.8%) without drugs administration (e.g., pain drugs, antibiotics, anti-hemorrhagics, or others) before (76.8%) or after (64.1%) the procedure. Only a little proportion of infertility centers included in the analysis proposes formally the ES as an add-on procedure (9.3%), even if, when proposed, the full description of the indications, efficacy, safety, and costs is never addressed. However, the overall information quality of the websites was generally “poor” ranging from 3 to 8 and having a low total score (4.7 ± 1.6; mean ± standard deviation).

**Conclusions:**

In Italy, ES is a procedure still performed among fertility specialists for improving the implantation rate in IVF patients. Moreover, they have a poor attitude in proposing ES as an add-on in the commercial setting.

## Background

Endometrial scratching (ES) or injury is intentional damage to the endometrium performed to improve reproductive outcomes of infertile women desiring pregnancy [1, 2]. To date, there is no adequate scientific evidence to suggest or recommend performing ES in clinical practice. Available data demonstrate that the effect of ES in improving implantation rates and fertility is small [1]. This is true for infertile patients scheduled for a first in vitro fertilization (IVF) cycle [3] and for unselected infertile patients who received an IVF treatment [4]. No clear efficacy of the ES has been demonstrated in infertile couples with unexplained or anovulatory infertility, who received intrauterine insemination (IUI) and ovulation inductors followed by free or timed sexual intercourses [5]. Two recent randomized controlled trials (RCTs) showed that ES does not improve the reproductive outcomes in infertile patients with polycystic ovary syndrome (PCOS) who receive fertility drugs [6] or with unexplained infertility [7] who had regular intercourses.

The main contrasting data regards the use of ES in IVF populations with recurrent or previous implantation failure [4, 8–14]. In fact, in a recent systematic review with metanalysis no effect of ES was observed in women suffering from recurrent or previous implantation failure [4]. Different from previous aggregate data [8, 9], that systematic review [4] restricted the analysis to studies judged at low risk of bias and included only eight among 35 published RCTs potentially eligible for meta-analysis. Notably, only two studies [10, 11] on women with recurrent implantation failure were included, while the majority of available RCTs on recurrent or previous implantation failure [12–15] were excluded.

Only a few trials have formally assessed the short and long-term complications of the procedure, including the maternal and fetal/neonatal safety after ES. In two recent RCTs [6, 7] on patients scheduled for free sexual intercourse, the ES performed during the early follicular phase using pipelle induced pain extremely variably, with an adjusted mean difference of about 3 on a 10-point pain scale. Bleeding is reported in patients who undergo ES in a variable proportion and may consist of minimal spotting for a few days in about half of cases [1, 16]. However, the collection and reporting of data on adverse events are generally poor, and in almost all systematic reviews it was not possible to synthesize quantitative data about pain and bleeding after the ES procedure [1, 16].

Based on these considerations, the present study was aimed to assess the current view and behavior towards ES among fertility specialists throughout infertility centers in Italy, and the relationship between these views and the attitudes towards using ES as an add-on in their commercial setting.

## Methods

The current study was aimed to answer two specific research questions: “what are clinicians’ perceptions and use of ES in Italy today?” and “how well do healthcare provider websites in Italy inform patients of the ES procedure?. To reply to the first question an online survey was performed using a detailed questionnaire, whereas an analysis of the websites of participating infertility centers was used to define whether these perceptions correspond or did not to the dissemination of the procedure in the commercial setting.

### Online survey

In February 2022, a detailed questionnaire was prepared by first author (SP) and revised and approved by the Board of the Italian Society of Human Reproduction (SIRU). It included 45 questions with the possibility to give “closed” multi-choice answers for 41 items and “open” answers for 4 items (Table 1). No other structured or semi-structured interviews were scheduled at study design.

**Table 1:** Characteristics of the physicians who completed and resent the questionnaire

Physicians’ characteristic	Number	Percentage (%)
**Sex**		
Male	26	60.5
Female	17	39.5
**Age**		
Less than 25	0	0
25- 45 years	18	41.9
46–65 years	24	55.8
More than 65	1	2.3
**Specialty**		
Clinician	43	100
Biologist	0	0
Other	0	0
**Academic qualification**		
Full Professor	1	2.3
Associate Professor	4	9.3
Adjunct Professor	1	2.3
Researcher	1	2.3
No academic role	36	83.7
**Role in the infertility center**		
Chief/Director	16	37.2
Attending physician	22	51.2
Other^a^	5	11.6

Data expressed as number and percentage (%). A total of 43 physicians have been included in the analysis

^a^External consultants

The questionnaire was composed of different items aimed at assessing several areas regarding the physicians’ perception about the use of ES in the clinical practice (Table 2). The questionnaire and a cover letter were sent to the SIRU secretariat for dissemination. In the cover letter the aim of the survey was explained, and it was asked to complete the questionnaire for each infertility center. Using the institutional email, the cover letter and the questionnaire were sent to all specialists in human reproduction affiliated with the SIRU. To increase the response rate, the specialists who replied to the email were invited as co-authors after having satisfied the international authorship criteria.

**Table 2:** Questions were made to physicians about ES and answers were sent by email. At total of 43 questionnaires were included in the analysis

1. How many patients with reproductive problems do you visit on average in a year?	
□ < 10	0/43; 0%
□ 10–100	5/43; 11.6%
□ 100–200	8/43; 18.6%
□ 200–300	8/43; 18.6%
□ 300–500	10/43; 23.3%
□ > 500	12/43; 27.9%
2. How many patients with reproductive problems do you treat on average in a year?	
□ < 10	0/43; 0%
□ 10–100	11/43; 25.6%
□ 100–200	6/43; 13.9%
□ 200–300	8/43; 18.6%
□ 300–500	8/43; 18.6%
□ > 500	10/43; 23.3%
3. Do you know the endometrial scratching procedure?	
□ yes, very well	34/43; 79.1%
□ yes, moderately well	9/43; 20.9%
□ no, little	0/43; 0%
□ no, very little	0/43; 0%
4. How do you rate your knowledge about endometrial scratching (from 0 to 10, also use decimals)?	
	8.0 (range, 5–10)
5. What percentage of the patients you treat do endometrial scratching?	
□ < 5%	18/43; 41.9%
□ 5–10%	12/43; 27.9%
□ 10–20%	9/43; 20.9%
□ 20–30%	2/43; 4.6%
□ 30–50%	2/43; 4.6%
□ > 50%	0/43; 0%
6. The patients with reproductive problems that you treat in one year suffer from (one or more):	
□ tubal factor	38/43; 88.4%
□ recurrent miscarriage	20/43; 46.5%
□ anovulation	26/43; 60.5%
□ PCOS	34/43; 79.1%
□ previous implantation failure	38/43; 88.4%
□ male factor	40/43; 93.0%
□ other (please specify):	
- Unexplained infertility	16/43; 37.2%
- Endometriosis/adenomyosis	8/43; 18.6%
- Reduced ovarian reserve	7/43; 16.3%
- Thin endometrium	2/43; 4.6%
- Fertility preservation	1/43; 2.3%
7. Which procedure do you use? (one or more)^a^	
□ Novak/vabra	6/39; 15.4%
□ pipelle	24/39; 61.5%
□ curette	1/39; 2.6%
□ hysteroscopy	19/39; 48.7%
□ other (please specify):	
- IUI catheter	2/39; 5.1%
8. Where do you do endometrial scratching? (one or more)^a^	
□ medical clinic	22/39; 56.4%
□ surgical outpatient clinic	15/39; 38.5%
□ operative room	5/39; 12.8%
9. Do you provide pre-established written informed consent form for your patients?^a^	
□ yes	20/39; 51.3%
□ no	19/39; 48.7%
10. Do you give oral informed consent to your patients?^a^	
□ yes	37/39; 94.9%
□ no	2/39; 5.1%
11. How do you motivate the patient to perform endometrial scratching? (one or more)^a^	
□ better rates of "babies in arms"	3/39; 7.7%
□ better implantation rates	31/39; 79.5%
□ better pregnancy rates	4/39; 10.3%
□ other (please specify):	
- To confirm a chronic endometritis	2/39; 5.1%
- To perform a “trial transfer”	1/39; 2.6%
12. What are the indications that you believe and validate endometrial scratching? (one or more)^a^	
□ no specific indication	7/39; 17.9%
□ repeated implantation failures	34/39; 87.2%
□ recurrent miscarriages	4/39; 10.3%
□ suboptimal endometrium during preparation for "frozen" transfer cycles	12/39; 30.8%
□ PCOS	4/39; 10.3%
□ use of drugs that have an endometrial “impact”	5/39; 12.8%
□ other (please specify):	
- Unexplained infertility	1/39; 2.6%
- Psychological indication	3/39; 7.7%
13. How much does endometrial scratching cost the patient (in euros)?^a^	
- Direct costs:	85 (range, 0–300)
- Indirect costs:	60 (range, 10–100)
14. When do you usually perform endometrial scratching?^a^	
□ in proliferative phase	9/39; 23.1%
□ in secretive phase	27/39; 69.2%
□ in a variable manner	3/39; 7.7%
15. How long before do you perform endometrial scratching in a fresh IVF cycle?^a^	
□ the cycle before stimulation	36/39; 92.3%
□ two cycles before stimulation	3/39; 7.7%
□ other (please specify):	0/39; 0%
16. How long before do you perform endometrial scratching in a "frozen" transfer cycle?^a^	
□ the cycle before endometrial preparation	36/39; 92.3%
□ two cycles before endometrial preparation	3/39; 7.7%
□ other (please specify):	0/39; 0%
17. How long before do you perform endometrial scratching in a stimulated cycle with free or timed intercourses?	
□ the cycle before stimulation^b^	20/20; 100%
□ two cycles before stimulation^b^	0/20; 0%
□ other (please specify):	
- I don’t perform ES in that clinical case	23/43; 53.5%
18. How long before do you perform endometrial scratching in a stimulated cycle with intrauterine insemination?	
□ the cycle before stimulation^b^	21/21; 100%
□ two cycles before stimulation^b^	0/21; 0%
□ other (please specify):	
- I don’t perform ES in that clinical case	22/43; 51.2%
19. How long before do you perform endometrial scratching in an ovulatory cycle with intrauterine insemination?	
□ the cycle before stimulation^b^	19/19; 100%
□ two cycles before stimulation^b^	0/19; 0%
□ other (please specify):	
- I don’t perform ES in that clinical case	24/43; 55.8%
20. How many times do you think endometrial scratching should be done?^a^	
□ only once before all treatments	17/39; 43.6%
□ before any reproductive attempt	19/39; 48.7%
□ other (please specify):	
- I don’t know	2/39; 5.1%
- According to histology	1/39; 2.6%
21. What are the greatest benefits of endometrial scratching in your clinical practice? (one or more)^a^	
□ better rates of "babies in arms"	1/39; 2.6%
□ better implantation rates	28/39; 71.8%
□ better pregnancy rates	4/39; 10.3%
□ other (please specify):	
- No benefit	9/39; 23.1%
- Psychological	3/39; 7.7%
- To reduce ET difficulty	1/39; 2.6%
22. What are the risks of endometrial scratching in your clinical practice? (one or more)^a^	
□ bleeding	11/39; 28.2%
□ pain	22/39; 56.4%
□ infection	2/39; 5.1%
□ other (please specify):	
- Nothing	13/39; 33.3%
23. Do you administer drugs before endometrial scratching?^a^	
□ always	6/39; 15.4%
□ never	30/39; 76.9%
□ sometimes	3/39; 7.7%
24. What do you give before endometrial scratching? (one or more)^a^	
□ pain drugs	9/39; 23.1%
□ anti-hemorrhagics	0/39; 0%
□ antibiotics	5/39; 12.8%
□ other (please specify):	
- Nothing	28/39; 71.8%
- Anti-spastic/myolitics	3/39; 7.7%
- Vaginal antiseptics	1/39; 2.6%
25. What do you prescribe after endometrial scratching? (one or more)^a^	
□ pain drugs	9/39; 23.1%
□ anti-hemorrhagics	0/39; 0%
□ antibiotics	8/39; 20.5%
□ other (please specify):	
- Nothing	25/39; 64.1%
- Vaginal antiseptics	1/39; 2.6%
26. Is there written consent for endometrial scratching in the center where you carry out the IUI and/or IVF/ICSI procedures?	
□ yes	18/43; 41.9%
□ no	25/43; 58.1%
27. In the center where the IUI and/or IVF/ICSI procedures are carried out, is there a written and shared diagnostic-therapeutic assistance pathway to carry out endometrial scratching?	
□ yes	9/43; 20.9%
□ no	34/43; 79.1%
28. In the center where the IUI and/or IVF/ICSI procedures are carried out, is there a written procedure for performing endometrial scratching?	
□ yes	13/43; 30.2%
□ no	30/43; 69.8%
29. According to recent literature, endometrial scratching should be recommended in case of (one or more):	
□ no specific indication	21/43; 48.8%
□ repeated implantation failures	20/43; 46.5%
□ recurrent miscarriages	3/43; 7.0%
□ during endometrial preparation in frozen cycles	3/43; 7.0%
□ PCOS	0/43; 0%
□ use of drugs with endometrial impact	3/43; 7.0%
□ other (please specify):	0/43; 0%
30. According to recent literature, endometrial scratching should be suggested in case of (one or more):	
□ no specific indication	12/43; 27.9%
□ repeated implantation failures	30/43; 69.8%
□ recurrent miscarriages	6/43; 13.9%
□ during endometrial preparation in frozen cycles	5/43; 11.6%
□ PCOS	2/43; 4.7%
□ use of drugs with endometrial impact	2/43; 4.7%
□ other (please specify):	
- Unexplained infertility	1/43; 2.3%
31. Based on the recent literature, it should be recommended/suggested not to perform endometrial scratching in case of (one or more):	
□ no specific indication	20/43; 46.5%
□ repeated implantation failures	0/43; 0%
□ recurrent miscarriages	1/43; 2.3%
□ during endometrial preparation in frozen cycles	2/43; 4.7%
□ PCOS	2/43; 4.7%
□ use of drugs with endometrial impact	1/43; 2.3%
□ endometritis	18/43; 41.8%
□ coagulation problems and/or bleeding diathesis	16/43; 37.2%
□ vaginismus	7/43; 16.3%
□ other (please specify):	
- First IVF-ET cycle	2/43; 4.7%
32. In what percentage do the patients who are offered endometrial scratching agree to perform it?	
□ < 20%	8/43; 18.6%
□ 20–30%	1/43; 2.3%
□ 30–40%	3/43; 7.0%
□ 50–60%	5/43; 11.6%
□ 60–70%	0/43; 0%
□ > 70%	26/43; 60.5%^c^
33. What percentage of patients who are offered endometrial scratching already know the procedure?	
□ < 20%	22/43; 51.2%
□ 20–30%	5/43; 11.6%
□ 30–40%	4/43; 9.3%
□ 50–60%	7/43; 16.3%
□ 60–70%	1/43; 2.3%
□ > 70%	4/43; 9.3%
34. What percentage of patients spontaneously propose/require endometrial scratching?	
□ < 20%	23/43; 53.5%
□ 20–30%	10/43; 23.3%
□ 30–40%	4/43; 9.3%
□ 50–60%	5/43; 11.6%
□ 60–70%	0/43; 0%
□ > 70%	1/43; 2.3%
35. Do you believe that performing endometrial scratching is a free choice of the patient?	
□ yes	10/43; 23.3%
□ no	33/43; 76.7%
36. What are the types of patients who spontaneously propose endometrial scratching (one or more)?	
□ in the first IVF cycle	1/43; 2.3%
□ in the second IVF cycle	9/43; 20.9%
□ repeated implantation failure	35/43; 81.4%
□ recurrent miscarriages	12/43; 27.9%
□ unexplained infertility	6/43; 14.0%
□ patients with unexplained infertility scheduled to cycles of ovulation induction plus free/timed intercourses	0/43; 0%
□ patients with unexplained infertility scheduled to cycles of ovulation induction plus IUI	0/43; 0%
□ unexplained infertility subjected to cycles of free / timed intercourses	1/43; 2.3%
□ unexplained infertility subjected to cycles of IUI	1/43; 2.3%
□ PCOS-related anovulatory infertility	0/43; 0%
□ infertility and PCOS	0/43; 0%
□ anovulatory infertility and PCOS scheduled to ovulation induction cycles	0/43; 0%
□ no specific indication	6/43; 14.0%
□ other (please specify):	
- Not specified	1/43; 2.3%
37. Do you believe that endometrial scratching improves reproductive outcomes in patients (one or more)?	
□ in the first IVF cycle	1/43; 2.3%
□ in the second IVF cycle	4/43; 9.3%
□ repeated implantation failure	31/43; 72.1%
□ recurrent miscarriages	4/43; 9.3%
□ unexplained infertility	9/43; 20.9%
□ patients with unexplained infertility scheduled to cycles of ovulation induction plus free/timed intercourses	3/43; 7.0%
□ patients with unexplained infertility scheduled to cycles of ovulation induction plus IUI	3/43; 7.0%
□ unexplained infertility scheduled to cycles of free/targeted intercourses	2/43; 4.7%
□ unexplained infertility subjected to cycles of IUI	2/43; 4.7%
□ PCOS-related anovulatory infertility	1/43; 2.3%
□ infertility and PCOS	2/43; 4.7%
□ anovulatory infertility and PCOS scheduled to ovulation induction cycles	0/43; 0%
□ no specific indication	10/43; 23.3%
□ other (please specify):	
- Thin endometrium	1/43; 2.3%
- Obese patients	1/43; 2.3%
38. What feature do you consider most to suggest/recommend endometrial scratching (one or more)?	
□ Repeated implantation failure	30/43; 69.8%
□ Nothing	11/43; 25.6%
□ Thin/suboptimal endometrium	4/43; 9.3%
□ Suspect of chronic endometritis	2/43; 4.7%
□ Patients’ request	2/43; 4.7%
□ Unexplained infertility	1/43; 2.3%
□ Trial transfer	1/43; 2.3%
□ Reproductive history	1/43; 2.3%
39. Over the last five years, the number of your patients who have undergone endometrial scratching is:	
□ reduced	10/43; 23.3%
□ increased	18/43; 41.9%
□ stable	15/43; 34.9%
40. Over the last five years, the number of patients who come to your clinic/center and have undergone endometrial scratching is:	
□ reduced	11/43; 25.6%
□ increased	19/43; 44.2%
□ stable	13/43; 30.2%
41. According to recent literature, the effectiveness of endometrial scratching (one or more):	
□ improves reproductive outcomes in first cycle IVF/ICSI patients	0/43; 0%
□ improves reproductive outcomes in second-cycle IVF/ICSI patients	1/43; 2.3%
□ improves reproductive outcomes in patients with repeated implantation failure	22/43; 51.2%
□ improves reproductive outcomes in patients with recurrent miscarriages	3/43; 7.0%
□ improves reproductive outcomes in patients with unexplained infertility	8/43; 18.6%
□ improves reproductive outcomes in patients with unexplained infertility undergoing ovulation induction cycles	1/43; 2.3%
□ improves reproductive outcomes in patients with unexplained infertility undergoing cycles of ovulation induction and IUI	2/43; 4.7%
□ improves reproductive outcomes in patients with unexplained infertility undergoing timed intercourse cycles	0/43; 0%
□ improves reproductive outcomes in patients with unexplained infertility undergoing free intercourse cycles	0/43; 0%
□ improves reproductive outcomes in patients with PCOS anovulatory infertility	0/43; 0%
□ improves reproductive outcomes in patients with infertility and PCOS	0/43; 0%
□ improves reproductive outcomes in patients with anovulatory infertility and PCOS undergoing ovulation induction cycles	0/43; 0%
□ does not improve reproductive outcomes in any type of patient studied	20/43; 46.5%
□ other (please specify):	
- Thin endometrium	1/43; 2.3%
42. Based on recent literature on the safety of endometrial scratching (one or more):	
□ it is a totally safe technique	22/43; 51.2%
□ its short-term safety is unknown	1/43; 2.3%
□ its long-term safety is unknown	2/43; 4.7%
□ its short- and long-term safety is unknown	0/43; 0%
□ it is associated with small blood losses	16/43; 37.2%
□ it is associated with pain	12//43; 27.9%
□ it is not associated with adverse events	11/43; 25.6%
□ other (please specify):	
- Potential pelvic infections	2/43; 4.7%
- Potential uterine perforations	1/43; 2.3%
43. On a scale of 0 to 10, how effective do you consider endometrial scratching (use also decimals)?^d^	
	4.0 (range, 0–9)
44. On a scale of 0 to 10, how safe do you consider endometrial scratching (use also decimals)?	
	7.95 (range, 3–10)
45. On a scale of 0 to 10, how easy do you consider endometrial scratching (use also decimals)?	
	8.5 (range, 5–10)

Specific items were used to assess the number of patients with reproductive problems visited and treated on average in a year per center (items 1 and 2), the reproductive problems of the patients managed (item 6), the personal knowledge of the ES (items 3 and 4), the percentage of the patients treated with ES (item 5), the specific tool used for ES (item 7), the setting where the procedure is done (item 8), the use of a specific written and/or oral informed consent (items 9 and 10), the presence of a specific written consent (item 26) and a shared diagnostic-therapeutic assistance pathway (including a written procedure) (items 27 and 28) in the referral infertility centers, the indications to perform ES (item 12), how the patients are motivated to perform it (item 11), the costs of the procedure (item 13), the timing of the procedure according to the cycle phases (item 14) and in different clinical scenarios (items 15–19), the potential “duration” of the effect of the procedure (item 20), its potential benefits (item 21) and risks (item 22), the drugs given/prescribed before (items 23 and 24) and after (item 25) the procedure, the potential recommendations or suggestions to perform (items 29 and 30) and to not perform (item 31) the procedure according to recent scientific evidence, the proportion of patients who accept to receive or do not receive the procedure (item 32), who already know the procedure (item 33), who spontaneously require the procedure (item 34), and their potential reasons (item 36). Were also investigated the opinion about the ethic to perform the procedure at patient request (item 35), the personal indications to perform ES according to patients’ characteristics/features (items 37 and 38), the changes (if any) in the proportion of patients who have undergone ES during the last five years (items 39 and 40), the quantification of the perceived effectiveness (item 43), safety (item 44), and feasibility (item 45) of the procedure

Data are expressed as proportion or median (range, min–max). The proportion is calculated for each answer on a total of 43 physicians from different referral infertility centers

^a^data obtained considering 39 physicians from different referral infertility centers because in 4 cases the physician declared to do not perform ES

^b^data obtained considering only physicians who use ES in that case

^c^In case of physicians who did not perform ES, the question was: “In what percentage do the patients who are not offered endometrial scratching agree to do not perform it?”

^d^data calculated considering 42 physicians because in one case the answer was “I don’t know”

A deadline of two weeks was given to obtain the highest possible response rate; a web link with the electronic access to the survey was published on the social media of the scientific society and sent to the different national WhatsApp chats of SIRU subscribers. A second email was sent after the first week.

Respondents were able to reply only once including name, surname, email address, and affiliation. The email address of the SIRU was used to answer any questions or comments from participants. Only one questionnaire was considered for each center to avoid data duplication. For each questionnaire, missing data/values were allowed. Questionnaires missing in more than three questionnaire items were considered invalid and excluded by analysis.

### Analysis of the websites of referral infertility centers

Using a predefined electronic chart, two different online searches were also performed about the different infertility centers included in the survey. The first was performed using the website of the Italian Institute for Health (IIH, or Istituto Superiore della Sanità, ISS) at https://w3.iss.it/site/registropma/pub/centri/centripma.aspx to define their characteristics, including their geographic location, the type of center (private, public, or national health service affiliated center), the specific treatments performed (IVF, non-IVF cycles, fresh/frozen cycles, gamete donation, and so on), and the number of treatments. The second online search was made on the websites of each center to define the presence and the quality of information for clinicians and/or for patients about add-ons treatments with regard for the ES, including data on efficacy, safety, and costs. A treatment was considered as an add-on procedure if reported with an amber or red symbol according to Human Fertilisation and Embryology Authority (HFEA) (http://www.hfea.gov.uk/treatments/treatment-add-ons/).

The quality of information about ES given on infertility centers websites was assessed using a scoring matrix [17] modified for ES (Table 3). It included ten specific questions (scored from 0 to 2 points), and the possible scores ranged from 0 to 13 points. Information recorded from each infertility center website was classified as ‘excellent’ if the score was 9 points or more, ‘moderate’ if the score was between 5 and 8, and ‘poor’ if it was 4 points or less. Each online search was performed during March 2022 by two different authors (SP, SM) and then compared. Disagreements were resolved by re-checking the websites.

**Table 3:** Scoring system to assess information about ES given from each infertility center website

Question	Possible score
Is the name of the clinic clearly mentioned?	1 point
Is contact information given?	1 point
Is graphic explanation of the ES given?	1 point
Is an explanation of the safety of the ES given?	2 points
Are the source and date of the data accurately provided?	1 point
Are explanations of success rates given?	2 points
Are the data based on the clinic’s experience?	1 point
Are the success rates based on specific population?	1 point
Is the efficacy of conceiving after ES accurately stated according to the patient’s characteristics?	1 point
Is the cost of the procedure given?	2 points

Information was classified as ‘excellent’ if the score was 9 points or more, ‘moderate’ if the score was between 5 and 8, and ‘poor’ if it was 4 points or less

### Data analysis

At study design, no sample size was predefined and calculated since the study was not aimed to include neither hypothesis testing nor inferential statistics methods for quantitative analysis. Furthermore, no limit was given to our sample to be as representative as possible. Survey data included in the final analysis were tabulated, expressed as numbers and percentages or as median (range). The quality of information from websites of infertility centers with different characteristics were expressed as mean and standard deviation (SD) and compared using a one sample *t* test. The proportions of websites classified as ‘excellent’, ‘moderate’ and ‘poor’ were expressed as percentages and compared using the Chi square test. *P* values less than 0.05 were considered statistically significant.

### Ethics

The study protocol was in accordance with the Declaration of Helsinki. Participants’ consent was specifically requested and obtained by email before data analysis. Participants were informed that the confidentiality and security of their data will be assured. The study fulfilled General Data Protection Regulation (GDPR) requirements, including the processing, storage, and protection of all data. However, the study did not involve humans and/or the use of human tissue and/or hospital records samples, and no patients’ data were recorded and analyzed. The ethical approval was granted by the Ethical Committee of Catania (Italy)**.** All data regarding the infertility centers / physicians were anonymized before manuscript submission and no identifiable information was submitted/published.

## Results

### Data from online survey

Survey invitations were sent to all SIRU-affiliated physicians (n. 344). Forty-five questionnaires were completed and re-emailed to the SIRU secretariat. Considering a total of 71 SIRU-affiliated infertility centers, the response rate was of 60.6% (43 questionnaires / 71 infertility centers). All included questionnaires were completed in their entirety. Two questionnaires were excluded because sent in both cases by two different physicians from the same infertility center. The corresponding author contacted by email the two physicians to obtain only one document signed by both. Thus, a total of 43 questionnaires (corresponding to 43 infertility Centers) out of 45 received were included in the final analysis.

Table 1 details the main characteristics of the physicians who completed and re-sent the questionnaire. The results of the survey are summarized in Table 2. The physicians had a good experience in human reproduction because they self-reported to visit and treat more than 200 patients yearly in a large proportion of cases (69.8%). Their clinical experience in infertility treatment was wide including female and male infertility, and other specific fertility problems, including endometriosis/adenomyosis, unexplained infertility, and fertility preservation. Their ES personal knowledge was largely considered very good and at least moderately good in almost all cases (100%) with an average score of about 8.

The proportions of infertile patients who receive ES was reported to be very small from physicians resulting to be performed in less than 10% in most of cases (69.8%). In 4 cases, the physicians declared to not perform the ES in their clinical practice. The procedure was mainly performed using pipelle (61.5%) and through hysteroscopy (48.7%) in a medical ambulatory (56.4%). Of particular interest are data regarding informed consent. In slightly more than half of the cases (51.3%), a pre-defined written informed consent was employed, whereas oral informed consent was given in almost all cases (94.9%). Similarly, an available specific written consent, a shared diagnostic-therapeutic assistance pathway, and a written procedure were available in a little proportion of the Infertility Centers.

In a large proportion of cases, the physician reported to perform ES for treating the repeated implantation failure (87.2%) and to obtain a better implantation rate (79.5%) was considered the main reason to perform ES.

The direct cost of the procedure was extremely variable especially in private settings where it ranged from no cost for 4 physicians (it was considered included in the IVF cycle when indicated) to 300 euros when included in the hysteroscopic procedure. On the other hand, in public and affiliated infertility centers the cost was partially covered by National Health Service and varied according to different Italian Regions (from 30.4 euros to 37 euros).

The ES is considered to perform in the secretory phase in most cases (69.2%). However, when associated with hysteroscopy, the physicians reported to prefer the proliferative phase of the cycle. In a very high percentage of cases (92.3%), the ES was scheduled at the cycle before the IVF procedure. The ES is considered to be performed only once before all treatments in about a half of cases (43.6%), whereas in another half of cases it is considered to be performed before any reproductive attempt (48.7%).

An improved implantation rate was considered the greatest benefit of ES in clinical practice (71.8%), even if physicians did not believe in its clear reproductive benefit. Even if in a little proportion of cases (7.7%), psychological reasons (to increase patients’ motivation) were also considered a benefit of performing ES. On the other hand, in about one-third of cases, the ES is considered without risks, and the pain and the bleeding are not considered the main risk of the procedure in large percentage of cases.

In a very high proportion of cases (71.8%), ES is performed without drugs administration before and after procedure. However, pain drugs (23.1%) and antibiotics (20.5%) are considered the most used compounds both before and after the treatment. A high proportion of physicians (46.5%) declared that, according to recent literature, the ES should be recommended and suggested in case of repeated implantation failure. Moreover, in another high percentage (48.8%), the physicians replied that there is no specific indication to recommend or suggest ES. No specific contraindication is considered by many physicians (46.5%) following recent data, and the main reasons not to perform an ES are the suspected endometritis (41.8%) and the coagulation problems and/or bleeding diathesis (37.2%).

More than one-fifth of the involved physicians believed that receiving ES is a free choice for the patients and that ES improves reproductive outcomes in patients with repeated implantation failure and/or unexplained infertility. Repeated implantation failure was the main clinical feature to suggest/recommend ES (69.8%), and it was reported that, according to recent literature, ES improves reproductive outcomes in patients with repeated implantation failure (68.9%). However, in about a quarter of cases, no specific indication was believed to be valid or to follow, and about a half of the physicians believe that ES does not improve reproductive outcomes in any specific patient studied. Over the last five years, the number of patients who have undergone ES is considered increased (44.2%) or stable (30.2%). Finally, the ES is generally considered not very effective (score 4.0, range 0–9), but safe (score 7.95, range 3–10) and feasible (score 8.5, range, 5–10).

### Data from the websites of referral infertility centers

Table 4 details the characteristics of the infertility centers included in the final analysis. The centers resulted equally distributed over the Italian territory, homogeneous for the type of infertility center (public, private or IIH-affiliated), including several infertility procedures, and with a good number of infertility treatments performed each year.

**Table 4:** Characteristics of the 43 infertility centers included in the study. Data recorded from the website of the Italian Institute of Health (IIH) at https://w3.iss.it/site/registropma/pub/centri/centripma.aspx

**Characteristic**^a^	**n. (%) or median (range)**
**Geographic distribution**	
North Italy	19 (44)
Central Italy	5 (12)
South Italy	19 (44)
**Type of center**	
Private centers	23 (53)
Public centers	16 (37)
IIH-affiliated centers	4 (9)
**Treatments performed**	
IVF/ICSI and IUI cycles	31 (72)
IUI cycles alone	5 (12)
Others^b^	7 (16)
GD cycles	
IVF/ICSI and IUI cycles	19 (44.2)
IUI cycles alone	2 (4.7)
**Number of treatments performed yearly**	
IVF/ICSI cycles	181 (4—1224)
IUI cycles	54 (1—362)
GD cycles	71 (0—246)
Others^c^	77.5 (0—813)

*ET* embryo transfer, *GD* gamete donation, *IUI* intrauterine insemination, *ICSI* intracytoplasmic sperm injection, *IVF* in vitro fertilization

^a^Last official data available (2019)

^b^Infertility diagnosis, ovulation monitoring, and so on

^c^Frozen ET and IVF/ICSI cycles from frozen oocytes

A high proportion of infertility centers included in the analysis had a website (38/43, 88%), and specific web pages for add-ons were detected in about one third of cases (11/38, 28.9%). ES procedure was proposed on the websites of only few infertility centers (4/38, 10.5%), and almost in all cases, the precise description of the technique (2/4, 50%), its potential efficacy (2/4, 50%), indications (3/4, 75%), contraindications (0/4, 0%), and costs (0/4, 0%) were poorly detailed.

The information quality scores from Italian infertility centers ranged from 3 to 8, and the total mean score (± SD) for all websites was 4.7 ± 1.6. In no case the websites were defined ‘excellent’, whereas they resulted ‘moderate’ and ‘poor’ in 4 and 7 cases, respectively. No difference among private, public, and IIH-affiliated fertility centers for different geographic areas and among volumes of services was detected about the quality of the information of the websites (data not shown).

## Discussion

HFEA has included the ES among several treatment adjuncts, also known as add-ons. This means that ES should not be recommended for routine use because there is conflicting evidence about its efficacy in improving the chances of having a baby for most fertility patients and further research is required (http://www.hfea.gov.uk/treatments/treatment-add-ons/). This is true in consideration of the recent available clinical evidence [1, 16]. However, ancillary treatments/procedures to improve the success rate of infertile patients scheduled for IVF and/or non-IVF cycles are widespread and growing more and more. This is likely associated with a significant financial burden [18].

Current data show that Italian fertility specialists believe that only a small proportion of their infertile patients receive ES, and mainly before IVF cycles in case of repeated implantation failure. The procedure is performed using pipelle during the secretory phase of the cycle in a high proportion of cases in a medical ambulatory setting and may achieve high costs especially in private settings. Interesting, about a quarter of physicians report no clear benefit of the ES considering their clinical practice and available scientific evidence, and this procedure is proposed in a small proportion of infertility centers’ websites.

Two previous surveys [19, 20] of fertility specialists in Australia, New Zealand, and the United Kingdom (UK) reported a substantial reduction of the ES in IVF cycles after negative results from large RCT [10]. Moreover, a very high proportion of fertility specialists still recommended ES believing that the procedure increases the probability of success in case of repeated implantation failure [19, 20].

New scientific evidence [1, 16] seemed to influence only a little proportion of fertility specialists in our study, whereas in other Countries [20] this influence achieves about half of fertility specialists. Even if ES is performed in a low proportion of well-selected cases, the incidence of physicians who use ES is very high and a large proportion of fertility specialists still offer ES in Italy, especially in women with recurrent implantation failure believing that it is an effective procedure to improve the pregnancy and live birth rates. The use of ES is also enhanced by the consideration that it is a procedure highly safe and feasible, also in the medical ambulatory settings. Our data on ES safety are not in agreement with previous survey data [20] probably because a different proportion of laboratory scientists and clinicians was included or because the Italian fertility specialists are less inclined to change therapeutic strategies. The analysis of our findings demonstrates that the risks and disadvantages of the procedure are not perceived and considered negligible by survey participants. On the contrary, pain/discomfort and risk of infection associated with the procedure were reported, respectively, in 86% and 60% of the fertility specialists [20]. Unfortunately, adverse events of the ES generally are poorly collected and re-ported in the literature. ES is associated with pain and induces bleeding in a high proportion of cases [3, 4, 6, 7]. However, in IVF patients, severe pain was reported in less than 3% of cases and the bleeding consists of minimal spotting in more than half of cases [16]. Of particular interest is the absolute lack in literature of long-term safety data [16].

In patients scheduled for free sexual intercourses, the ES, performed during the early follicular phase, induced a pain about 3 points higher in pain score [6, 7]. However, the ES performed during the early follicular phase was associated with fewer pain scores than during the luteal phase [5].

Contrarily to previous data on the use of IVF add-ons [21], no difference in the proportion of ES use was observed between private and public/IIS-affiliated centers. The decision to perform ES may be due to a real belief that the procedure is effective in sell-selected patients or to the need to suggest something different for a clinical condition highly frustrating for patients and doctors. The use of ES with “psychological indication” has been reported in our and previous studies [19, 20].

Of note, our data demonstrate that in any case, the participants refer to formal documents to avoid ES in the clinical practice; even in a discrete proportion of cases performing an ES “at the patient’s request” is considered ethical. On the contrary, previous data showed that 20% of fertility centers in Australia, New Zealand, and the UK have a clear and official policy of not offering or recommending ES to any patient in their clinic [20].

From a pharmaco-economic point of view, our data are in line with previous data about the direct costs of the procedure [20, 22]. In a private setting, ES performed through hysteroscopy can cost up to 300 euros, and if repeated before each procedure or every 3–6 months may significantly influence the total cost of the infertility treatment. Furthermore, contrary to previous data [22], only a little proportion of responders reported indirect costs considering the ES as an office-based procedure non-associated with indirect costs in a high proportion of cases.

Our study has several strengths. First, the current is one of the few published studies [19, 20] exploring the view and the attitudes of specialists in human reproduction about the use of ES in the clinical practice. Second, the participants were all Italians, and no language barrier was present. Third, fertility specialists, as well as the infertility Centers, were well distributed across the Italian Regions. Four, the data analysis included SIRU-affiliated specialists in human reproduction with “potential” good knowledge of ES and a high volume of treatments yearly. Even if more than 80% of the physicians did not have an academic role, they were Chief/Director of the referral infertility Center in more than one third of cases.

The present study has also limitations mainly due to the low proportion of Italian infertility Centers included in the study. The response to the invitation was of 60.6%, and it could probably have been greater. The use of non-anonymous questionnaires could have an influence, and the request to sign and to make identifiable the questionnaires might be a barrier to response. However, this has probably improved the quality of the answers and the overall attention to the questionnaire completion. To this regard, no questionnaire was excluded from the analysis due to sub-optimal completion. The use of incentives, and not the anonymity, seems to be not an effective strategy to increase the response rate [23]. Our study design included the potential authorship to the manuscript as incentive to respond, and this has probably increased the response rate [23]. Finally, the restriction of the study to SIRU-affiliated physicians/centers may have introduced a bias making the findings not completely generalizable to other settings.

Current findings obtained by analyzing the specific web pages of the referral infertility Centers are not in agreement with previous studies showing as many infertility Centers in the UK, in Australia and New Zealand, and in general on the web, offer a range of treatments in addition to standard IVF procedures [24–26]. Only a little proportion of Italian fertility Centers offer add-ons procedures for improving the success chances in addition to standard IVF/ICSI procedures, and fewer ES is offered to patients. However, when offered, in none of the cases the potential benefits are well detailed and quantified according to the available evidence, as previously shown [24–26]. Similarly, there is no case where the potential risks and/or side effects and/or adverse events are specified, whereas the costs are rarely reported. Current data on the information quality given on the infertility centers websites indicate that women did not receive the information they need to make well-informed choices. These findings are in line with a previous study [27] in which the quality of the information of the Australian and US websites about elective oocyte cryopreservation was analyzed using the same tool [17] resulting “poor” in a very high proportion of cases. In this regard, it is well known that infertile couples scheduled for reproductive treatments are not well informed about the risks of the treatment, and that the success rates of treatments are often overestimated [28, 29]. Well-balanced, simplified but realistic information regarding not only the potential beneficial effects but also the potential consequences of the intervention, declaration about conflicts of interest, alternative interventions, potential harms, and costs should be available [25].

Recent interesting and provocative data [30, 31] highlighted a worldwide decline in live birth rates in IVF cycles during the last years and suggested as potential cause the introduction of new add-ons to IVF practice, including the use of “all-freeze” cycles, mild ovarian stimulation protocols, preimplantation genetic testing for aneuploidy and increasing utilization of elective single embryo transfer, and so on. Thus, we should be honest with our patients about the lack of evidence and either not offer the ES or offer it only as part of a research project [32]. We should explain to them that we cannot offer these treatments as there is no evidence of benefit and there is generally little evidence-based safety data [1]. Moreover, infertile patients are very vulnerable and probably are very susceptible to the “hope technology” [33]. In many cases of unexplained unsuccess also clinicians want to create opportunity for success using this approach [34].

Since the first study on ES published by Barash et al. in 2003 [35], several RCTs have failed to give a definitive answer to the question whether ES may im-prove the IVF outcome in specific IVF subpopulations [36–38]. Waiting for further well-done clarifying studies [1], we agree [32] to recommend to our patients that their financial resources are better saved for additional cycles of evidence-based treatments at lower costs instead of inflating the costs of a single cycle.

## Conclusions

The findings from this semi-quantitative analysis provide insights into experience in and preference for ES of the Italian specialists of reproductive medicine. Present data show that there is a lack of complete knowledge regarding ES that is still performed as an add-on procedure for improving reproductive outcomes, especially in IVF patients with recurrent implantation failure or for improving implantation rates. Italian fertility specialists consider the procedure cheap, simple, feasible, and safe. Fortunately, both fertility specialists and clinical centers seem to have a poor attitude towards ES as an add-on in their commercial setting, and this notwithstanding in Italy there is not any regulation of the fertility add-on treatments for IVF and non-IVF procedures. More transparency and information on the ES are probably needed on the fertility centers’ websites to improve well-informed treatment decisions to perform or not to perform the procedure. In consideration of the lack of clear clinical evidence about its efficacy and long-term safety, collaborative networks are probably needed to include clinical data of patients who receive ES in the National register to monitor the risk/effective ratio of this still not completely known procedure.

## Data Availability

All data generated or analyzed during this study are included in this published article. The raw data analyzed during the current study are available from the corresponding author on reasonable request, and not publicly available to protect the privacy of the centers involved.

## Abbreviations

ES: Endometrial scratching
ET: Embryo transfer
GD: Gamete donation
GDPR: General Data Protection Regulation
HFEA: Human Fertilisation and Embryology Authority
ICSI: Intracytoplasmic sperm injection
IVF: In vitro fertilization
IUI: Intrauterine insemination
IIH: Italian Institute for Health
SIRU: Italian Society of Human Reproduction
PCOS: Polycystic ovary syndrome
RCT: Randomized controlled trial
UK: United Kingdom
